# CRISPR/Cas9_-3NLS_/sgHMGA2@PDA nanosystem is the potential efficient gene editing therapy for gastric cancer with HMGA2 high expression

**DOI:** 10.3389/fonc.2022.978533

**Published:** 2022-09-02

**Authors:** Zhouying Wu, Xue Huo, Tingyu Yang, Kun Liu, Ting Wu, Zongqi Feng, Min Wang, Feng Li, Jianchao Jia, Xiaoran Zhang, Wenming Gao, Lan Yu

**Affiliations:** ^1^ Clinical Medical Research Center/Inner Mongolia Key Laboratory of Gene Regulation of the Metabolic Diseases, Inner Mongolia People’s Hospital, Hohhot, China; ^2^ College of Agronomy, Inner Mongolia Agricultural University, Hohhot, China; ^3^ Departments of Cardiology, Hohhot First Hospital, Hohhot, China; ^4^ Department of Endocrine and Metabolic Diseases, Inner Mongolia People’s Hospital, Hohhot, China

**Keywords:** PDA self-polymerization, RNP delivery, gastric cancer, CRISPR/Cas9_-3NLS_/sgHMGA2@PDA, gene therapy

## Abstract

Gene therapy is one of the target therapies with promising clinical use for gastric cancer (GC). However, the delivery of the CRISPR/Cas9/sgRNA (RNP) gene editing tool severely limits the practical therapeutic effect of GC. Therefore, it is a great challenge to develop an RNP delivery system that is simple to prepare and can rapidly encapsulate RNP while achieving high delivery and gene editing efficiency. We developed, for the first time, the CRISPR/Cas9@PDA nano-delivery system that can achieve high-efficiency delivery (95%) of CRISPR/Cas9_-3NLS_/sgHMGA2 and high-efficient HMGA2 gene editing (82%) of GC cells. In particular, the experiment’s weak alkaline environment can not only protect the activity of CRISPR/Cas9_-3NLS_/sgHMGA2 but also trigger the self-polymerization of polydopamine (PDA). Meanwhile, the presence of KE in the CRISPR/Cas9 amino acid sequence can achieve the directional growth of PDA, thus forming a core–shell structure that protects CRISPR/Cas9_-3NLS_/sgHMGA2. This efficient CRISPR/Cas9_-3NLS_/sgHMGA2 delivery and HMGA2 gene editing ability has also been verified in mice, which can significantly inhibit tumor growth in mice. The success of building the delivery system and its ideal treating effect give hope to the efficacious treatment for the GC patients with HMGA2 high expression.

## Introduction

Although gastric cancer (GC) does not rank the top on the cancer morbidity and mortality list, it has been one of the highlighted malignant tumors because of its low 5-year survival rate (1). The prognosis of several cancers such as non-small cell lung cancer has benefited a lot since the research of precision treatment reached an upper ladder (2, 3). However, besides the traditional treatment methods, the target therapy in GC is not as optimistic as other cancers are (4, 5). For one thing, fewer genes for treating GC can be targeted; for another, less clinical target therapy can be available. Therefore, to develop a novel, efficacious, low-toxicity target treating method needs to be solved urgently.

For years, the methods of cancer targeting therapy have sprouted, and a lot of target medicines have been invented for clinical use (6). Among these methods, gene therapy has magnetized the attention of researchers for its unparalleled specificity and sensitivity (7). For treating cancer, the core is editing the target gene *via* either gene transfer or gene knockout techniques. Actually, tumor-related gene therapy accounts for more than half of the gene therapies entering the clinical stage (8). Whether gene editing is conducted successfully or not will depend on two key points: one is that the targetable gene is precisely found out, and the other is that the gene-editing tool will not work until it is transported by a certain delivery system to the right place where it should work. Thus, it is of great significance to find suitable gene targets and develop a delivery system with efficient delivery of gene editing tools, especially the development of delivery systems.

In the previous research, we have proved that HMGA2 was overexpressed in most types of GC and positively related with the GC development and the patients’ prognosis (9). It was also found that HMGA2 was involved in multiple key signaling pathways in GC development (10, 11). Interestingly, HMGA2 plays a key role during embryonic development and shows a high expression physiologically, while it is absent or expressed in the extremely low level in human normal tissue after birth (12). Such expression characteristics of HMGA2 make it an ideal target to precisely strike GC without hurting the normal tissue.

The CRISPR/Cas9 system has become a promising gene editing tool because of its predominant gene editing ability (13). The sequence of the single-guide RNA (sgRNA) was designed beforehand according to the sequence of the target gene. The designed sgRNA is joined with CRISPR/Cas9, being the “eye” of CRISPR/Cas9, which will lead CRISPR/Cas9 to the target sequence to fulfill the task (14). More researchers prefer to choose CRISPR/Cas9 plus the designed sgRNA to develop the gene therapy tool than other methods, and three forms have been invented: plasmid DNA (pDNA), messenger RNA (mRNA), and ribonucleoprotein (RNP) (15). Most of pDNA and mRNA do not work well as expected because intracellular transcription and translation are needed. RNP is the complex of CRISPR/Cas9 protein with sgRNA. Compared to pDNA and mRNA, RNP functions as soon as it enters the nucleus of target cells, and such instant gene editing is the base of the high efficiency (16). Although RNP has already shown the promising application prospects, its high negative charge and large size make it difficult to be protected from degradation or denaturation during preparation and delivery procedure (17). In particular, due to the presence of various enzymes during *in vivo* delivery, RNP is easily degraded, leading to lower gene editing efficiency (18).

To date, several non-viral nanocarriers have been reported for RNP delivery *in vitro*, including inorganic nanocarriers (19–23), DNA nanoclews (24), and polymeric nanocarriers (25, 26). Most methods utilize electrostatic interactions, usually adsorbing RNP after nanoparticle formation, and this simple interaction results in the easy shedding of RNP during delivery and its vulnerability to enzymes *in vivo*, which reduces the delivery efficiency. In order to overcome the problem of low RNP delivery efficiency caused by surface adsorption. Researchers have developed a variety of delivery methods for RNP encapsulated in lipid nanoparticles (15, 27–29). This structure of encapsulating RNP inside can well avoid enzymatic degradation *in vivo* and achieve delivery of RNP. However, the preparation of lipid nanoparticles is very complicated, which leads to the expensive price, which is not conducive to the promotion of actual clinical treatment (15). Meanwhile, the formation of most lipid nanoparticles is carried out in an acidic environment, and the presence of an acidic environment is prone to denaturation of RNP, resulting in low gene editing efficiency for lipid nanoparticle delivery (29). Therefore, it is a great challenge to develop an RNP delivery system that is simple to prepare and can rapidly encapsulate RNP while achieving high delivery efficiency and high gene editing efficiency.

In recent years, inspired by the adhesion of marine mussels, a protein that endows mussels with super-adhesive properties, mussel adhesion proteins, has been discovered in the byssus of mussels (30). Inspired by this, its derivative polydopamine (PDA) has received widespread attention. The surface of PDA is rich in functional groups such as catechol and has strong adhesion, so it has been widely used as a coating layer on the surface of various materials (31). PDA can undergo self-polymerization under weak alkaline conditions and can adhere to the surface of nanoparticles (32) or bacteria (33) to form a core–shell structure, which can prevent nanoparticles from being oxidized or prevent bacteria from being affected by the external environment. Most importantly, PDA possesses outstanding biocompatibility, biodegradation, and extremely low toxicity (30, 34). Actually, PDA has been approved as a medical material by the US FDA (35). Based on this, PDA has been widely used in the field of biomedicine and has shown good application prospects.

**Figure 1:**
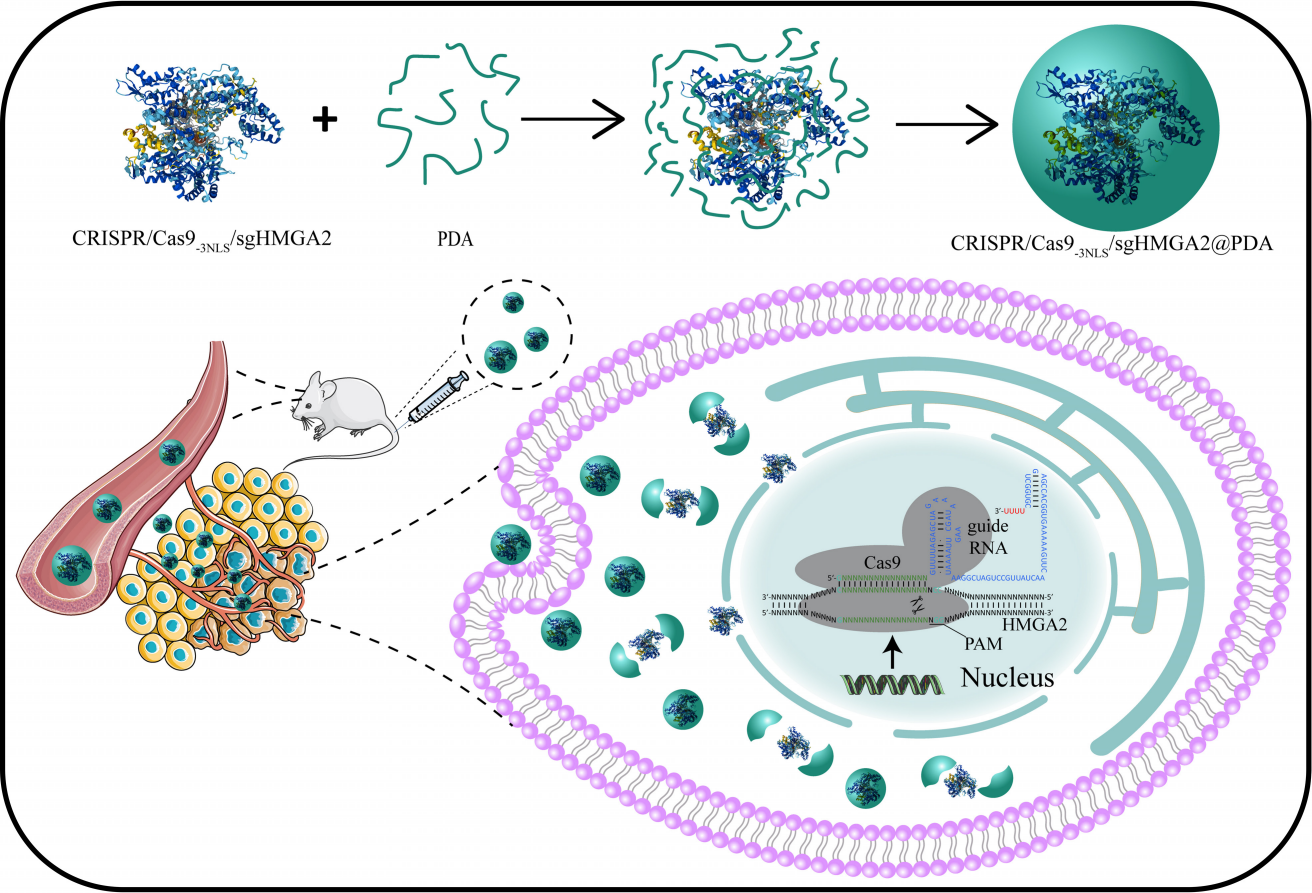
Design of the CRISPR/Cas9_-3NLS_/sgHMGA2@PDA delivery system, and its intracellular delivery pathways.

In this study, we develop for the first time a CRISPR/Cas9@PDA nano-delivery system that can achieve high-efficiency delivery of CRISPR/Cas9_-3NLS_/sgHMGA2 and high-efficiency HMGA2 gene editing of gastric cancer (**Figure 1**). This encapsulates CRISPR/Cas9_-3NLS_/sgHMGA2 with simple and rapid PDA self-polymerization in a weak alkaline environment which could keep the viability of CRISPR/Cas9_-3NLS_/sgHMGA2 and also provide the engine condition for PDA. CRISPR/Cas9_-3NLS_/sgHMGA2@PDA can achieve efficient delivery of CRISPR/Cas9_-3NLS_/sgHMGA2 with a maximum delivery efficiency of 95% and can quickly decompose and release CRISPR/Cas9_-3NLS_/sgHMGA2 when entering gastric cancer cells, achieving efficient HMGA2 gene editing in gastric cancer cells, with an editing efficiency of 82%. Meanwhile, the high delivery and gene-editing efficiency were clearly illustrated in the mouse experiments. The sizes of the tumors in the CRISPR/Cas9_-3NLS_/sgHMGA2@PDA-treating group were much smaller than the ones in the control group. Therefore, the CRISPR/Cas9_-3NLS_/sgHMGA2@PDA delivery system can achieve desired CRISPR/Cas9_-3NLS_/sgHMGA2 delivery efficiency and HMGA2 gene editing ability *in vitro* and *in vivo* to treat GC with high expression of HMGA2.

## Materials and methods

### Materials

3-(4,5-Dimethyl-2-thiazolyl)-2,5-diphenyl-2-H-tetrazolium bromide (MTT), dopamine hydrochloride (DA, H8502-25G), sodium bicarbonate (NaHCO_3_, 792519), and isopropyl β-D-1-thiogalactopyranoside (IPTG, I6758) were brought from Sigma-Aldrich (St. Louis, MO, USA). Alexa Fluor 488 carboxylic acid NHS ester (succinimidyl ester) (A20000) and Pierce Protease Inhibitor Mini Tablets EDTA Free (A32955) were bought from Thermo Fisher. Imidazole (56750-100G) was brought from MERCK, and 4% paraformaldehyde (4% PFA) (SL1830) was obtained from Coolaber. 6-Diamidino-2-phenylindole (DAPI) (D4080) was bought from US EVERBRIGHT. Dimethyl sulfoxide (DMSO) and ethanol were purchased from Sinopharm Chemical Reagent Co., Ltd. Phosphate-buffered saline (PBS, pH 7.4 and 5.0), fetal bovine serum (FBS), DMEM, penicillin–streptomycin, and trypsin-EDTA were purchased from Gibco Life Technologies. Enzymes were obtained from NEB. All other chemicals used in this study were purchased from Sigma-Aldrich in analytical reagent grade and used without further purification. Ultrapure water (18.25 MΩ.cm, 25°C) was used to prepare all solutions.

### Preparation of CRISPR/Cas9_-3NLS_ and CRISPR/Cas9_-3NLS-AF488_

The CRISPR/Cas9_-3NLS_ expression vector was conducted *via* introducing the nucleotide sequence encoding two additional SV40 nuclear localization signals (NLSs) into the CRISPR/Cas9 coding region of the backbone sequence of the pET28a/Cas9-Cys vector (Addgene, Plasmid 53261), at the terminus of the nucleotide sequence that encodes the native NLS (PKKKRKV) and that before of the stop codon (TAA). Briefly, the specific pair primers (N3-F/R), at the 5′ end of which a nucleotide sequence that can separately encode two NLSs was contained, were designed, and the sequences are shown in **Table 1**. The PCR amplification was performed by using the high-fidelity enzyme POD-plus-neo (KOD-401, Toyobo, Japan) which has excellent elongation capability, and a 9,646-bp PCR product was purified by using QIAquick PCR Purification Kit (Qiagen). After it was phosphorylated and self-ligated, the conducted vector was subcloned into *E.coli* DH5α (Code No. 9057, TAKARA), and the colony PCR and Sanger sequence were performed to identify the CRISPR/Cas9_-3NLS_ expression vector.

**Table 1:** Sequences of primers and sgRNA used in the study.

Gene	Forward (5′ to 3′)	Reverse (5′ to 3′)
N3-F/R	TACCTTTCTCTTCTTTTTTGGTACTTTTCTCTTTTTCTTTGGAGGTCCGGA	CCAAAAAAGAAGAGAAAGGTACCAAAAAAGAAGAGAAAGGTATAAGCGGCCGCACTCGAGCAC
h-HMGA2-2F/h-HMGA2-1R	ACGTCCGGTGTTGATGGTG	GAGAAAAACGGCCAAGAGGC
h-HMGA2-2F/h-HMGA2-R	ACGTCCGGTGTTGATGGTG	CTCCCTTCAAAAGATCCAACTG
h-HMGA2-2F/h-HMGA2-2R	ACGTCCGGTGTTGATGGTG	CCAGGAAGCAGCAGCAAGA
M13F/M13R	TGTAAAACGACGGCCAGT	GGTCATAGCTGTTTCCT
**sgRNA**	**Forward (5′ to 3′)**	**Reverse (5′ to 3′)**
HMGA2-1	CACCTCCTCTCTTCTGAGGCGCTG	AAACCAGCGCCTCAGAAGAGAGGA
HMGA2-2	CACCGGTCCTCTCTTCTGAGGCGCT	AAACAGCGCCTCAGAAGAGAGGACC
HMGA2-3	CACCGTGGGGCGGCAGGTTGTCCCT	AAACAGGGACAACCTGCCGCCCCAC

The BL21(DE3)-Rosetta cell was transformed with the recombinant vector and was cultured in LB medium with 50 μg/ml kanamycin. Isopropyl-β-D-thiogalactopyranoside (IPTG, 0.5 mM) was added when the OD of 0.7–0.9 and the incubation were continued in the shaker at 30°C for 10 h. Then the cell was pelleted and lysed *via* sonication (45% power, 8-s pulse 8-s rest, 60 min), and the soluble fraction was transferred to a HisTrap HP column (GE, UK). The column was washed and equilibrated by distilled water and binding buffer (20 mM sodium phosphate, 0.5 M NaCl, 30 mM imidazole, pH 7.4) before loading the soluble fraction; after being twice washed with binding buffer, the target-induced CRISPR/Cas9_-3NLS_ was eluted with elution buffer (20 mM sodium phosphate, 0.5M NaCl, 250 mM imidazole, pH 7.4). Furthermore, the concentrations of purified protein were analyzed using SDS-PAGE and Coomassie brilliant blue and quantified with Pierce™ BCA Protein Assay Kit (Thermo#23225). The purified CRISPR/Cas9_-3NLS_ was labeled with AF-488 tag (Alexa-488, succinimidyl ester, Thermo Fisher, USA) following the instructions when necessary.

### sgHMGA2 design and synthesis

Three single-guide (sg) RNAs targeting the function AT-hook domain of HMGA2 were designed. The pDR274 vector (Addgene, 42250) was used to conduct the sgRNA expression plasmids: pDR274-sgHMGA2-1, pDR274-sgHMGA2-2, and pDR274-sgHMGA2-3. Briefly, three annealed oligonucleotides were inserted downstream the T7 promoter in the Bsal digested pDR274, respectively, and those conducted plasmids were transformed in E. coli DH5α followed by DNA which were extracted with a plasmid extraction kit (QIAGEN, 12143). After the digestion by endonuclease DraI (Thermo Fisher, 0224), the digested products (290 bp) were purified using the QIAquick PCR Purification Kit and were then subjected to *in vitro* transcription with MEGAscript™ T7 Transcription Kit (Thermo Fisher, K0441). The transcribed sgRNAs were extracted using TRIzol™ Reagent (Invitrogen, 15596018) and quantified by Nano2000 spectrophotometry (Thermo Fisher Scientific Inc., USA) and nucleic acid electrophoresis. The primer sequences used for sgRNA synthesis are listed in **Table 1**.

### Assemble and cleavage activity of CRISPR/Cas9_-3NLS_/sgHMGA2

In order to assemble the CRISPR/Cas9_-3NLS_/sgHMGA2 complex, the purified CRISPR/Cas9_-3NLS_ protein and sgHMGA2 (sgHMGA2-1: sgHMGA2-2: sgHMGA2-3 = 1:1:1) were incubated in 1× NEBuffer 2 for 30 min at 37°C with concentrations of 1.6 and 3.2 nM, respectively. Afterward, to verify the cleavage activity of CRISPR/Cas9_-3NLS_/sgHMGA2, we synthesized a DNA bearing a target sequence, which was added as a concentration of 150 nM, and the mixture was further co-incubated for 30 min under the same condition above. Then the proteinase K and RNAse A were added separately to remove the needless protein and RNA. Before analysis, we carried out 2% gel electrophoresis reaction with 2 μl 6× native loading buffer and 10 μl mixture after cleavage by CRISPR/Cas9_-3NLS_/sgHMGA2 to investigate cleavage activity.

### Preparation of CRISPR/Cas9_-3NLS_/sgHMGA2@PDA

The powdery DA was dissolved with sterile enzyme-free water as a concentration of 27 mg/ml. The pH of the prepared CRISPR/Cas9_-3NLS_/sgHMGA2 solution (pH 8.5) was adjusted using 0.1% NaHCO_3_. One hundred microliters of DA solution was taken with a concentration of 27 mg/ml, which was quickly added dropwise to a 1.5-ml centrifuge tube containing 300 μl of CRISPR/Cas9_-3NLS_/sgHMGA2 solution (160 nM) and vortexed rapidly until the solution turns black to stop the reaction to obtain CRISPR/Cas9_-3NLS_/sgHMGA2@PDA. After being synthesized, the coated CRISPR/Cas9_-3NLS_/sgHMGA2@PDA was rinsed with distilled water and centrifuged at 10,000 rpm for 10 min to obtain the precipitate containing CRISPR/Cas9_-3NLS_/sgHMGA2@PDA; the precipitate was finally washed and resuspended in the aqueous solution.

### Characterization of CRISPR/Cas9_-3NLS_/sgHMGA2@PDA

The electron micrograph (TEM) of CRISPR/Cas9_-3NLS_/sgHMGA2@PDA was obtained on a Hitachi H800 (Tokyo, Japan) transmission electron microscope with an operating voltage of 200 kV. The zeta potential of CRISPR/Cas9_-3NLS_/sgHMGA2 and CRISPR/Cas9_-3NLS_/sgHMGA2@PDA was characterized by Zetasizer Nano instrument (Malvern Nano ZS90, Malvern, UK).

### Cell viability and delivery system toxicity test

The toxicity of the CRISPR/Cas9_-3NLS_/sgHMGA2@PDA NP delivery system was assessed through cell proliferation measured by MTT assay. Briefly, MKN45 cells and MGC-803 cells were seeded in 96-well plates at 4 × 10^3^ and 1 × 10^3^ per well, respectively, and cultured overnight. The next day, the mediums were replaced with 0.2mL fresh mediums containing CRISPR/Cas9_-3NLS_/sgHMGA2@PDA at the concentration of 0, 80, 160, 240, 320nM of CRISPR/Cas9_-3NLS_. After 24 h of co-incubation, the cell viability was measured using CellTiter 96 Non-Radioactive Cell Proliferation Assay Kit (Promega, G4001).

### Intracellular and nucleus delivery of CRISPR/Cas9_-3NLS_/sgHMGA2@PDA

The intracellular and nucleus delivery efficiency of CRISPR/Cas9_-3NLS_/sgHMGA2@PDA was evaluated by confocal laser scanning microscopy (Leica TCS SP8) and flow cytometry (Beckman Coulter, Navios, USA) *via* tagging CRISPR/Cas9_-3NLS_ with AF-488 prior to the assembly of CRISPR/Cas9_-3NLS_/sgHMGA2. Briefly, MKN-45 and MGC-803 cells (2 × 10^4^ per well) were seeded in 24-well plates with climbing glasses and cultured overnight. Subsequently, cells were incubated with 500 μl of 10% FBS DMEM containing CRISPR/Cas9_-3NLS-AF-488_/sgHMGA2@PDA (CRISPR/Cas9_-3NLS_ concentration, 16 nM) for 24 h. Cells were subjected directly (without any further processing) to confocal laser scanning microscopy for detecting intracellular delivery efficiency. After washing with PBS and staining with nucleus dye (DAPI, 5 μg/ml), cells were subjected to confocal laser scanning microscopy for detecting nucleus delivery efficiency. After collecting and staining, cells were subjected to flow cytometry and the data were analyzed using Kaluza Analysis Software (version 2.1).

### *In vitro* evaluation of the gene cleavage efficiency of CRISPR/Cas9_-3NLS_/sgHMGA2@PDA

DNA was extracted from MKN-45 cells cocultured with CRISPR/Cas9_-3NLS_/sgHMGA2@PDA NPs for 24, 48, and 72 h, respectively, using standard procedures. After DNA extraction, 100 to 500ng of DNA was used to amplify the sgHMGA2 targeting region using a specific primer pair (**Table 1**). PCR products were cloned into a TA cloning vector (Thermo Fisher Scientific). Following bacterial transformation, individual colonies were subjected to sequence analysis and the data were analyzed using APE Software.

### *In vivo* evaluation of the tumor-inhibiting ability of CRISPR/Cas9_-3NLS_/sgHMGA2@PDA

The NOD/SCID female mice (6 to 7 weeks of age) were purchased from Charles River Laboratories (Beijing, China) and maintained under specific pathogen-free conditions with free access to autoclaved food water and bedding. All animals were group-housed, and experiments were conducted in strict adherence to approved Institutional Animal Care and Use Committee protocol. MKN-45 cells (2 × 10^6^ cells) were harvested and dispersed into 150 μl of ice-cold PBS solution and subcutaneously injected into the right flank region of the mice. MKN-45-bearing mice were randomly divided into two groups when the tumor volume reached about 70 mm^3^ and were intravenously injected *via* tail with CRISPR/Cas9_-3NLS_@PDA (800 nM CRISPR/Cas9_-3NLS_) and CRISPR/Cas9_-3NLS_/sgHMGA2@PDA (800 nM CRISPR/Cas9_-3NLS_ and 1600 nM sgHMGA2) once a week from the 14th day to the 28th day, respectively. The xenograft volume and the mouse weight were measured and calculated every week from the 21th day to the 56th day. Then the weight of mice in groups were counted, and the tumor size were calculated as volume = (length × width^2^)/2.

### Immunofluorescence staining

The tumor tissues isolated from the mice treated with CRISPR/Cas9_-3NLS_@PDA and CRISPR/Cas9_-3NLS_/sgHMGA2@PDA, respectively, were fixed for 24 h in PBS containing 4% paraformaldehyde at 4°C. The tissues were then washed in PBS, infiltrated with 10% (1.5 h), 20% (1.5 h), and 30% (1.5 h) sucrose, embedded in OCT compound on dry ice. Cryosections of tumors were obtained using Cryostat Microtome (CM3050S, Leica, Germany) and stored at -80°C until stained. For immunofluorescence (IF) staining, sections were stained with anti-HMGA2 (1:300 dilution) (CST, #5269S), then revealed by Goat anti-Rabbit secondary antibodies labeled with Alexa Fluor 488 (Invitrogen). After DAPI staining, slides were mounted by Mounting Medium Fluoromount-G™ and were dried. During staining steps, all slides were blocked with 1% Normal Goat Serum, 5% BSA, 0.1% NaN_3_, 0.2% gelatin, and 0.1% Tx-100. Images of tumor sections were acquired at 20× and 40× with the confocal laser scanning microscope.

## Results and discussion

### Preparation of CRISPR/Cas9_-3NLS_/sgHMGA2 and verification of its cleavage activity

**Figure 2:**
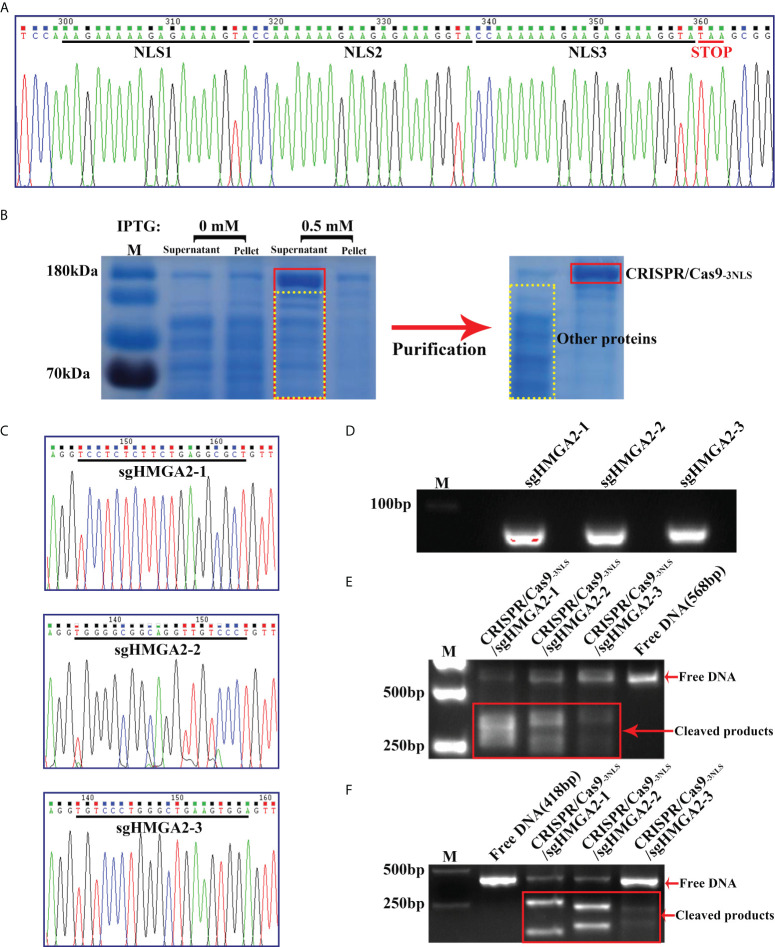
Preparation of CRISPR/Cas9-_3NLS_/sgHMGA2 and verification of its cleavage activity **(A)** The Sanger sequencing result of CRISPR/Cas9-_3NLS_; black lines indicate the bases encoding two inserted NLSs and one innate NLS, respectively; red line represents the stop code (TAA). **(B)** SDS-PAGE and Coomassie Brilliant blue staining for analyzing the induced expression and purification of CRISPR/Cas9-_3NLS_. **(C)** The Sanger sequencing results of the sgHMGA2 expression plasmid; black lines indicate the bases encoding sgHMGA2-1, sgHMGA2-2, and sgHMGA2-3, respectively. **(D)** The nucleic acid electrophoresis results of the purified sgHMGA2-1, sgHMGA2-2, and sgHMGA2-3. **(E, F)** The nucleic acid electrophoresis results of the fragments obtained from cleaving artificially synthesized double-stranded DNA by CRISPR/Cas9-_3NLS_/sgHMGA2; the length of DNA: 568 bp **(E)** and 418 bp **(F)**.

Naturally, one SV40 nuclear localization signal (NLS) exists near the C terminal of CRISPR/Cas9. To enhance the nuclear targeting power of CRISPR/Cas9, two additional NLS amino acid sequences were inserted beside the innate NLS in CRISPR/Cas9, forming CRISPR/Cas9_-3NLS_. Sanger sequencing showed that three NLSs were perfectly fixed in CRISPR/Cas9 (**Figure 2A**). Abundant CRISPR/Cas9_-3NLS_ was produced by the induced expression *via* IPTG in the BL21(DE3)-Rosetta cell, followed by purification through a HisTrap HP column (**Figure 2B**), making sure the adequate supply of CRISPR/Cas9_-3NLS_ in the whole experimental system. On the other side, three sgHMGA2 targeting the different sequences of HMGA2 were successfully integrated into the sgRNA expression plasmids. After the plasmids were proliferated in the E. coli DH5α, three kinds of sgHMGA2 were *in vitro* transcribed and purified and named as sgHMGA2-1, sgHMGA2-2, and sgHMGA2-3 (**Figures 2C, D**). Subsequently, CRISPR/Cas9_-3NLS_/sgHMGA2 was assembled *via* incubating the purified CRISPR/Cas9_-3NLS_ and sgHMGA2 (sgHMGA2-1: sgHMGA2-2: sgHMGA2-3 = 1: 1: 1) with concentrations of 1.6 and 3.2 nM, respectively. To confirm the cleavage activity of CRISPR/Cas9_-3NLS_/sgHMGA2, two double-stranded DNA with sgHMGA2-targeting sequences were artificially synthesized, respectively. This DNA was used as a verifying system and co-incubated with CRISPR/Cas9_-3NLS_/sgHMGA2 for 30 min. The DNA was cleaved into the fragments whose size was in line with the one we theoretically inferred (**Figures 2E, F**). These results indicated that CRISPR/Cas9_-3NLS_/sgHMGA2 exhibits superior gene editing activity.

### Synthesis and properties of CRISPR/Cas9_-3NLS_/sgHMGA2@PDA

**Figure 3:**
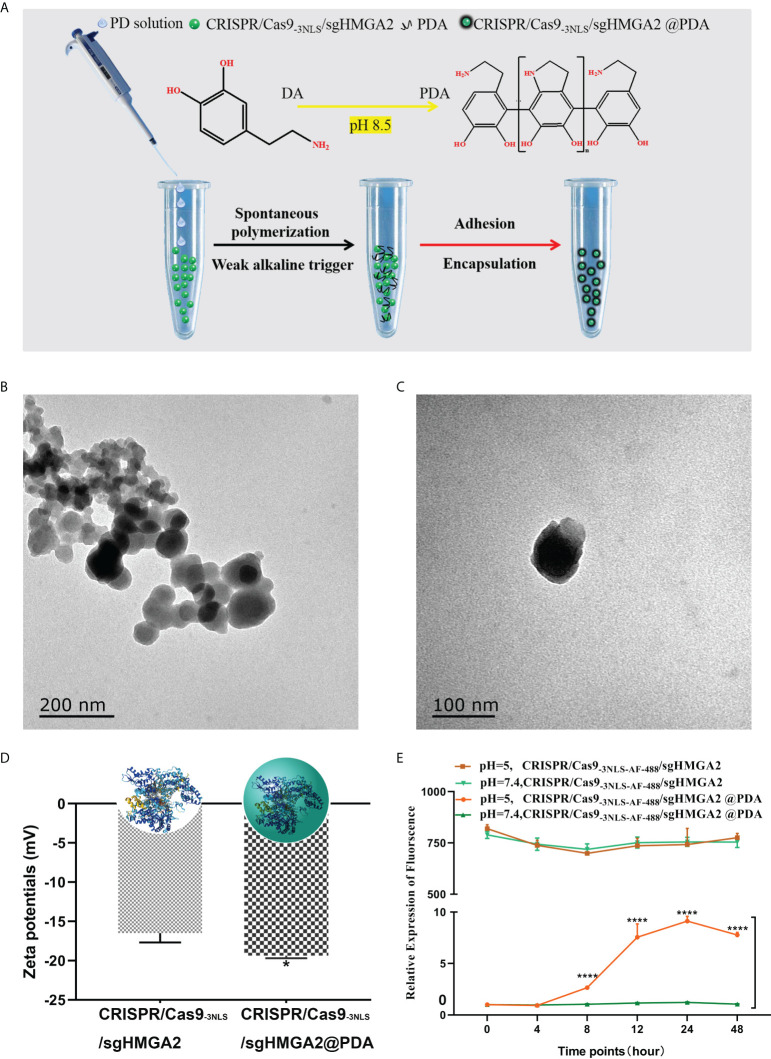
Synthesis and properties of CRISPR/Cas9_-3NLS_/sgHMGA2@PDA **(A)** The schematic synthesis of the CRISPR/Cas9_-3NLS_/sgHMGA2@PDA delivery system. **(B, C)** The TEM of CRISPR/Cas9_-3NLS_/sgHMGA2@PDA. **(D)** Zeta potentials of CRISPR/Cas9_-3NLS_/sgHMGA2 and CRISPR/Cas9-_3NLS_/sgHMGA2@PDA in water. **(E)** CRISPR/Cas9_-3NLS-AF-488_/sgHMGA2 release profiles of CRISPR/Cas9_-3NLS_/sgHMGA2@PDA in different pH environments. ****P < 0.001, Student’s t test. Error bars represent SD.

Inspired by mussel proteins, we exploited the properties of PDA to develop a simple and rapid method for encapsulating CRISPR/Cas9_-3NLS_/sgHMGA2. The schematic diagram of the formation of CRISPR/Cas9_-3NLS_/sgHMGA2@PDA is shown in **Figure 3A**. Firstly, CRISPR/Cas9_-3NLS_/sgHMGA2 solution (adjusted to pH 8.5 with NaHCO_3_) was added to the centrifuge tube. The abundant bicarbonate radical existing in this alkaline environment (pH 8.5) can maintain the distance among the molecules, protecting the activity of CRISPR/Cas9_-3NLS_/sgHMGA2 in the course of the reaction. Also, when the PD solution was quickly dropped into the CRISPR/Cas9_-3NLS_/sgHMGA2 tube, such environment could provide the necessary polymerizing condition for PD, resulting in the rapid self-polymerization of the PD to form PDA. Due to the existence of a large number of groups on the surface of PDA, especially the -NH_3_ and -COOH groups, PDA is prone to interact with the amino acid in the CRISPR/Cas9_-3NLS_ proteins. Adequate PDA adhered to the surface of CRISPR/Cas9_-3NLS_/sgHMGA2 and was encapsulated to form CRISPR/Cas9_-3NLS_/sgHMGA2@PDA with the core–shell structure due to the interacting force. As shown in **Figure 3B**, the size of the synthesized CRISPR/Cas9_-3NLS_/sgHMGA2@PDA is about 60–90 nm, while some of these smaller particles may be PDA nanoparticles formed due to PD self-nucleation. The core–shell structure can be clearly observed *via* the TEM image (**Figure 3C**) of the single CRISPR/Cas9_-3NLS_/sgHMGA2@PDA, and the outer layer is the PDA shell. As shown in **Figure 3D**, the zeta potential data of CRISPR/Cas9_-3NLS_/sgHMGA2 and CRISPR/Cas9_-3NLS_/sgHMGA2@PDA are significantly different, and the potential of the latter is lower, further indicating that the PDA successfully encapsulated CRISPR/Cas9_-3NLS_/sgHMGA2, causing the change in zeta potential. Therefore, we successfully constructed a simple and fast method to realize the formation of the CRISPR/Cas9_-3NLS_/sgHMGA2@PDA core–shell structures. Bergthold’s research found that PDA could interact with proteins to achieve an oriented growth of PDA (36), because of the presence of the amino acid sequence KE in the protein. **Figure S1** shows the amino acid sequence of the CRISPR/Cas9 protein in our synthesized CRISPR/Cas9_-3NLS_/sgHMGA2, and plenty of KE sequences can be found in the sequence. It is these KE which make the CRISPR/Cas9_-3NLS_/sgHMGA2 possess a large number of acting sites for the adhesion of PDA, thereby realizing the oriented growth of PDA and forming CRISPR/Cas9_-3NLS_/sgHMGA2@PDA core–shell nanostructures.

The stability of the CRISPR/Cas9_-3NLS_/sgHMGA2@PDA structure was further investigated *in vitro*. As shown in **Figure 3E**, CRISPR/Cas9_-3NLS_/sgHMGA2@PDA hardly released CRISPR/Cas9_-3NLS_/sgHMGA2 in PBS at pH 7.4. Only in a lower acidic environment (pH 5.0) can PDA degrade to release CRISPR/Cas9_-3NLS_/sgHMGA2, and the released amount was quite low. Therefore, the synthesized CRISPR/Cas9_-3NLS_/sgHMGA2@PDA nanostructures have good stability; thus, it will be storage and transportation friendly. Notably, the fluid environment in the human body is neutral, while the tumor microenvironment is an acidic environment (37). Therefore, CRISPR/Cas9_-3NLS_/sgHMGA2@PDA can protect CRISPR/Cas9_-3NLS_/sgHMGA2 well from being unshelled before reaching the tumor cells and can rapidly release CRISPR/Cas9_-3NLS_/sgHMGA2 after entering the tumor cells (**Supplementary Video 1**). The biosafety of a gene editing tool delivery system severely limits its application in the biomedical field. As shown in **Figure S2**, when CRISPR/Cas9_-3NLS_ was not connected to sgHMGA2, the synthesized CRISPR/Cas9_-3NLS_@PDA basically did not affect the proliferation of cells; even when the CRISPR/Cas9_-3NLS_ concentration was up to 320 nM which is twice higher than the maximum concentration reported (21), the inhibition rate of the cells was less than 10%. This indicates that the synthesized CRISPR/Cas9_-3NLS_@PDA has low biotoxicity and can be well applied to the delivery of CRISPR/Cas9_-3NLS_/sgHMGA2.

### Intracellular and nuclear delivery of CRISPR/Cas9_-3NLS_/sgHMGA2@PDA

In order to visualize the delivery efficiency of CRISPR/Cas9**_-_**_3NLS_/sgHMGA2@PDA to the cells, Alexa Fluor (AF)-488 (yellow) was used to tag CRISPR/Cas9_-3NLS_/sgHMGA2 enwrapped in the PDA shell; thus, CRISPR/Cas9**_-_**_3NLS-AF-488_/sgHMGA2@PDA was produced. The core–shell structure could also be reflected directly from the color change shown in **Figure S3**. The solution showed a complete yellow color after AF-488 was attached to CRISPR/Cas9**_-_**_3NLS_/sgHMGA2, whose color was from AF-488 itself (**Figure S3B**), while when CRISPR/Cas9**_-_**_3NLS-AF-488_/sgHMGA2 was wrapped by PDA, CRISPR/Cas9**_-_**_3NLS-AF-488_/sgHMGA2@PDA is formed; the yellow color disappeared, suggesting that the yellow RNP was covered by the PDA coat (**Figure S3A**). Further, such color change illustrated that CRISPR/Cas9**_-_**_3NLS-AF-488_/sgHMGA2 was enveloped by PDA in the core–shell structure rather than surface adsorption. Therefore, it could be considered that the nanoparticle synthesis and entrapment efficiency were particularly high.

**Figure 4:**
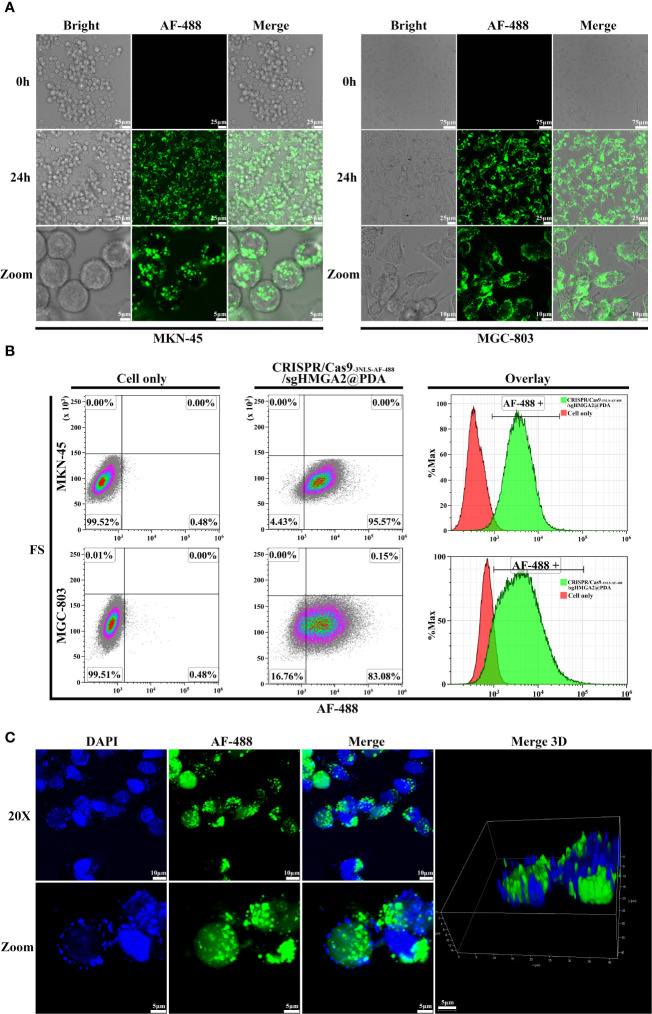
Intracellular and nucleus delivery of CRISPR/Cas9_-3NLS_/sgHMGA2@PDA **(A)** Intracellular delivery of CRISPR/Cas9_-3NLS_/sgHMGA2@PDA by confocal laser scanning microscopy. From the left to the right, combining images are MKN-45 and MGC-803 cells after co-incubation with CRISPR/Cas9_-3NLS_/sgHMGA2@PDA, respectively. First column of left image: bright-filled; second column: AF-488 fluorescence; third column: merged images. The upper line: 0 h after co-incubation, 20×; the middle: 24 h after co-incubation, 40×; the bottom: the magnified images of upper images. **(B)** Intracellular delivery of CRISPR/Cas9_-3NLS_/sgHMGA2@PDA by flow cytometry. The upper and bottom lines are MKN-45 and MGC-803 cells after co-incubation with CRISPR/Cas9_-3NLS_/sgHMGA2@PDA for 24 h, respectively; first column: cell only; second column: cells co-incubated with CRISPR/Cas9_-3NLS_/sgHMGA2@PDA; third column: overlay of the two images. AF-488 positive indicates the cells delivered successfully by CRISPR/Cas9_-3NLS_/sgHMGA2@PDA. **(C)** Nucleus delivery of CRISPR/Cas9_-3NLS_/sgHMGA2@PDA into MKN-45 cells by confocal laser scanning microscopy. First column: DAPI staining images; second column: AF-488 fluorescence images; third column: merged images of DAPI staining and AF-488 fluorescence; fourth column: 3D merged images of DAPI staining and AF-488 fluorescence; the upper: 24 h after co-incubation with CRISPR/Cas9_-3NLS_/sgHMGA2@PDA, 40×; bottom: the images zoomed from 40×.

Two kinds of human GC cells (MKN-45 and MGC-803) with an endogenous high expression of HMGA2 at four to five passages were co-incubated in the cell culture media with CRISPR/Cas9_-3NLS-AF-488_/sgHMGA2@PDA, respectively. As is shown in **Figure 4A**, at 0 h of incubation, neither green cells nor green fluorescence could be observed, suggesting that CRISPR/Cas9**_-_**_3NLS-AF-488_/sgHMGA2 was not adsorbed on the surface of PDA but integrally coated in the PDA shells of CRISPR/Cas9**_-_**_3NLS-AF-488_/sgHMGA2@PDA (**Figure 4A**, 0 h). At 24 h, the majority cells were green fluorescence positive. From the magnified pictures shown in the bottom line of **Figure 4A**, all the green fluorescence was gathered in the cell, indicating that CRISPR/Cas9**_-_**_3NLS-AF-488_/sgHMGA2@PDA was swallowed well and the PDA shells had been biodegraded after that in the cell. To verify the delivery efficiency of CRISPR/Cas9**_-_**_3NLS-AF-488_/sgHMGA2@PDA, the flow cytometry was used, and as shown in **Figure 4B**, the efficiency of intracellular delivery was 95% and 83% in MKN-45 and MGC-803 cells, respectively. Further, to observe the dynamic procedure of CRISPR/Cas9**_-_**_3NLS-AF-488_/sgHMGA2@PDA entering the cells, a constant observing video was shot for 24 h. The results showed that after the cells swallowed CRISPR/Cas9**_-_**_3NLS-AF-488_/sgHMGA2@PDA, it would take 6–8 h to produce the green fluorescence; the density of the green fluorescence increased and reached the climax at 24 h, that is, the PDA shells began to be degraded at the 6–8-h time points and digested almost completely at 24 h (**Supplementary Video 1**).

Furthermore, the low nucleus delivering efficiency of CRISPR/Cas9 ribonucleoprotein (RNP) has been an obstacle to conquer. In our experiments, because two additional NLSs were inserted in the CRISPR/Cas9 beforehand, which is mentioned above, the nucleus delivery efficiency was improved dramatically. As shown in 3D images (**Figure 4C**), after the cells were cultured in the media with CRISPR/Cas9**_-_**_3NLS-AF-488_/sgHMGA2@PDA for 24 h, the images clearly illustrated that CRISPR/Cas9_-AF-488_/sgHMGA2 entered the nuclei with high efficiency (**Supplementary Video 2**).

### The gene-cleaving effect of CRISPR/Cas9_-3NLS_/sgHMGA2@PDA *in vitro* and *in vivo*

Despite the high delivery efficiency of our vehicle, the high gene-cleaving effect should be most valued. In order to testify the efficiency of the gene editing, the cleaving capability of CRISPR/Cas9**_-_**_3NLS_/sgHMGA2@PDA was evaluated on MKN-45 cells and the animal model, respectively. The cells were co-incubated with CRISPR/Cas9**_-_**_3NLS_/sgHMGA2@PDA for 24, 48, and 72 h, while the cells with CRISPR/Cas9_-3NLS_@PDA were considered as the control group. Compared with the control cells, the cleavage efficiency of CRISPR/Cas9**_-_**_3NLS_/sgHMGA2@PDA was 68.9%, 80.55%, and 82% for 24, 48, and 72 h, respectively; as expected, the cleavage efficiency of the control group was zero. It is reported that a smaller DNA fragment is much easier to insert into the TA vector than the bigger one does. To observe the objective of our experiments, we calculated the fragment size inserted in the TA vector. Among the cleaved fragments reflected by the sequencing results of the TA colony PCR, taking 24 h for example, in the experimental group, 51 pieces of fragment were composed of 6 pieces ≦ 200 bp, 23 pieces ranging from 200 to 300 bp, and 22 pieces ≧ 300 bp. This phenomenon could also been seen in 48 and 72 h, indicating that our experimental system has a no small-fragment bias (**Table 2**).

**Table 2:** The cleavage efficiency calculation of CRISPR/Cas9-3NLS/sgHMGA2@PDA.

Time after co-incubation	Group	No. of total clones	No. of cleavage clones		No. of no cleavage clones	Cleavage rate (%)
24 h	CRISPR/Cas9-_3NLS_@PDA	20	0		20	0
	CRISPR/Cas9-_3NLS_/sgHMGA_2_@PDA	74	51	≦200 bp: 6	23	68.9
				200-300 bp: 23		
				≧300 bp: 22		
48 h	CRISPR/Cas9-_3NLS_@PDA	20	0		20	0
	CRISPR/Cas9-_3NLS_/sgHMGA_2_@PDA	36	29	≦200 bp: 4	7	80.55
				200-300 bp: 18		
				≧300 bp: 7		
72 h	CRISPR/Cas9-_3NLS_@PDA	20	0		20	0
	CRISPR/Cas9-_3NLS_/sgHMGA_2_@PDA	50	41	≦200 bp: 14	9	82
				200-300 bp: 16		
				≧300 bp: 11		

**Figure 5:**
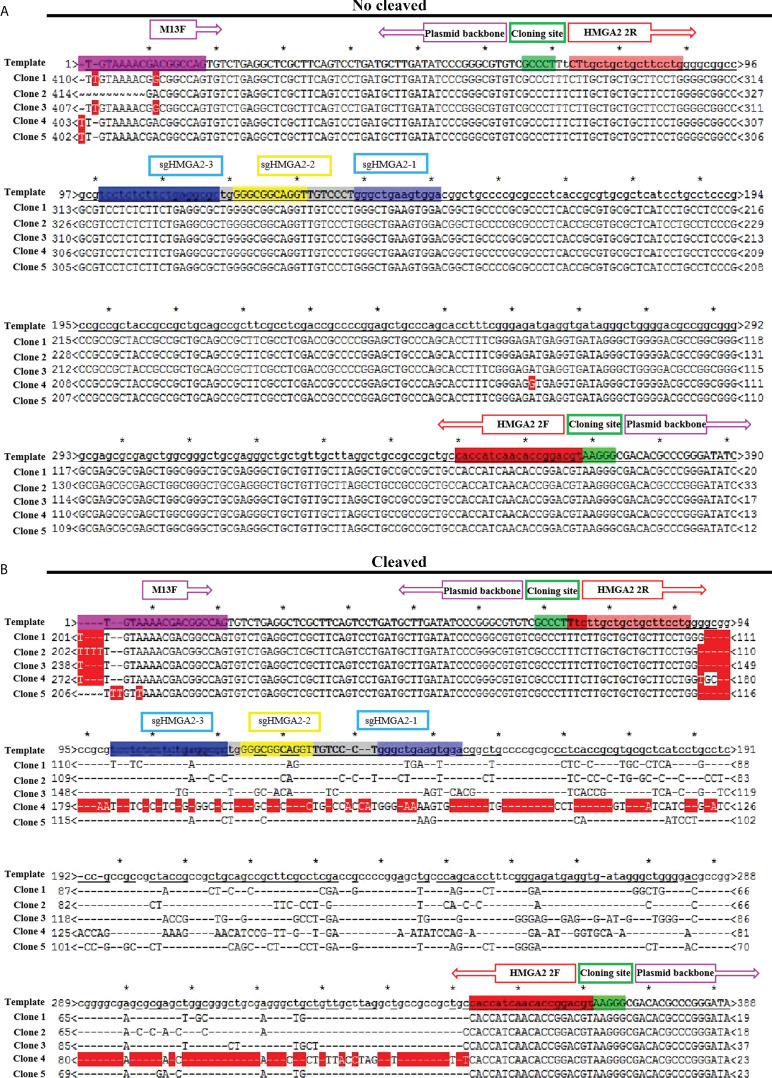
The gene-cleaving effect of CRISPR/Cas9_-3NLS_/sgHMGA2@PDA *in vitro* **(A)** Alignment of five representative colony PCR sequences derived from the cells co-incubated with CRISPR/Cas9_-3NLS_@PDA for 24 h to the supposed sequence. **(B)** Alignment of five representative colony PCR sequences derived from the cells co-incubated with CRISPR/Cas9_-3NLS_/sgHMGA2@PDA for 24 h to the supposed sequence; the region between the purple boxes represents the sequence of the plasmid backbone; the region between the red boxes represents the sequences of the inserted PCR fragment; green boxes: cloning site; the blue and yellow regions represent the sequence encoding sg-HMGA2-1, sg-HMGA2-2, and sg-HMGA2-3 *: the range between two asterisk represents 10bp.

When the cells were co-incubated with CRISPR/Cas9**_-_**_3NLS_/sgHMGA2@PDA, the DNA broke, HMGA2 in the genome was cleaved, and variable-size deletions occurred; thus, recombination of the DNA terminal happened. Sequencing the products from TA colony PCR can obtain the sequence of the cleavage. Two hundred twenty pieces of TA colony PCR were sequenced in our experiments. Here, five pieces from the control group and the experimental group, respectively, were picked out as examples to visualize the procedure. In the control group, sequencing results were totally the same as the supposed sequence, showing no cleavage (**Figure 5A**), whereas variable-size cleavage was shown by the sequencing in the experimental group (**Figure 5B**). In general, the activity of CRISPR/Cas9_-3NLS_/sgHMGA2 was well protected in this delivery system, and its high gene-editing efficiency was realized as high as 82%. The gene editing efficiency of CRISPR/Cas9_-3NLS_/sgHMGA2@PDA is higher than that of some other delivery systems reported in studies (**Table S1**), which may be due to the fact that the alkaline environment can better preserve the activity of CRISPR/Cas9_-3NLS_/sgHMGA2, and the existence of the core–shell structure can protect the CRISPR/Cas9_-3NLS_/sgHMGA2 well.

**Figure 6:**
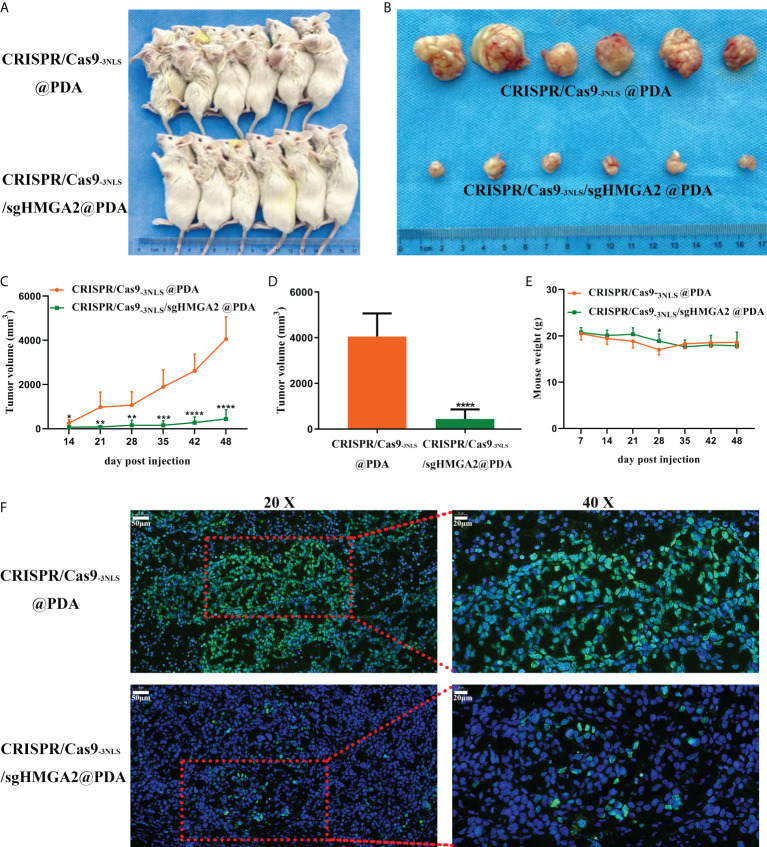
The gene-cleaving effect of CRISPR/Cas9_-3NLS_/sgHMGA2@PDA *in vivo* ***(*A, B*)*** The tumor-bearing NOD-SCID mice and the corresponding isolated tumors. The upper line: tumor-bearing mice injected with CRISPR/Cas9_-3NLS_@PDA (control); the bottom line: tumor-bearing mice injected with CRISPR/Cas9_-3NLS_/sgHMGA2@PDA. **(C)** The dynamic recording of the tumor volumes. The average of six tumors was calculated each week from the 14th day to the 48th day. **(D)** The average volume of six isolated tumors. **(E)** The dynamic recording of the mouse body weight. The average of six tumor-bearing mice was calculated each week from the 14th day to the 48th day. **(F)** Expression of HMGA2 in the tumors isolated from the two groups of mice. The upper line: tumor-bearing mice injected with CRISPR/Cas9_-3NLS_@PDA (control); the bottom line: tumor-bearing mice injected with CRISPR/Cas9_-3NLS_/sgHMGA2@PDA; the left column: ×20; the right column: images magnified from the red frame of the left side, ×40. Green: HMGA2-positive staining, blue: nuclei staining. *P<0.05, **P<0.01, ***P<0.001, ****P<0.0001. Student’s t test. Error bars represent SD.

Finally, the HMGA2-cleaving efficiency and tumor-inhibiting ability of CRISPR/Cas9_-3NLS_/sgHMGA2@PDA *in vivo* were evaluated in the tumor-bearing mouse models. The mice were randomly divided into two groups after the MKN-45 cells were injected for 2 weeks, and the tumorigenesis rate was 100%. CRISPR/Cas9_-3NLS_/sgHMGA2@PDA and CRISPR/Cas9_-3NLS_@PDA were intravenously injected *via* tail to the two groups, respectively. The injection was conducted once a week from the 14th day to the 28th day. The tumor volume was measured and calculated from the 14th day to the 48th day, and the mice were sacrificed at the 48th day. The antitumor effects of CRISPR/Cas9_-3NLS_/sgHMGA2@PDA are clearly shown in **Figure 6A, 6B**, and **6D**. Compared to the control group treated with CRISPR/Cas9_-3NLS_@PDA, the volumes of tumor, regardless of being carried on mice or isolated from mice, were significantly suppressed in the group treated with CRISPR/Cas9_-3NLS_/sgHMGA2@PDA. The concordant result could also be observed from the growth curves of the average tumor volumes of six mice, which were dynamically recorded and calculated each week (**Figure 6C**). Moreover, as is shown in **Figure 6E**, there was no significant change in the body weight of two groups of tumor-bearing mice which implies that CRISPR/Cas9_-3NLS_/sgHMGA2@PDA has low toxicity. In order to investigate whether HMGA2 was disrupted in the tumors isolated from the mice treated with CRISPR/Cas9-_3NLS_/sgHMGA2@PDA, IF staining was performed with the anti-HMGA2 antibody. HMGA2 IF stain positively (green) localized predominantly in the nucleus of the cells, as HMGA2 is a nuclear protein. The results showed that the expression of HMGA2 was decreased drastically in the edited group, indicating that the CRISPR/Cas9_-3NLS_/sgHMGA2@PDA NPs could disrupt HMGA2 vigorously *in vivo* as well (**Figure 6F**). Therefore, the experiments proved that CRISPR/Cas9_-3NLS_/sgHMGA2@PDA has magnificent delivery performance and excellent gene cleaving capacity both *in vivo* and *in vitro*. The obvious shrinking of the tumor, no weight loss, and good living status thoroughly proved that CRISPR/Cas9_-3NLS_/sgHMGA2@PDA could be a promising gene therapy tool with high performance and low toxicity in the future clinic use.

## Conclusion

In summary, we develop for the first time a CRISPR/Cas9@PDA nano-delivery system that can achieve high-efficiency delivery of CRISPR/Cas9_-3NLS_/sgHMGA2 and high-efficiency HMGA2 gene editing of gastric cancer. The CRISPR/Cas9_-3NLS_/sgHMGA2@PDA shows good stability and can exist stably *in vitro*. Meanwhile, CRISPR/Cas9_-3NLS_@PDA exhibits very low toxicity when no sgHMGA2 is present. *In vitro* experiments showed that CRISPR/Cas9_-3NLS_/sgHMGA2@PDA can achieve efficient delivery of CRISPR/Cas9_-3NLS_/sgHMGA2 and efficient HMGA2 gene editing in gastric cancer cells, with delivery and gene editing efficiencies as high as 95% and 82%, respectively. Finally, this high-efficiency delivery and gene editing efficiency can also be achieved in mice, which can significantly inhibit tumor growth in mice. This CRISPR/Cas9_-3NLS_/sgHMGA2@PDA delivery system shows good application prospects in gene-targeted therapy of gastric cancer.

## Data availability statement

The original contributions presented in the study are included in the article/**Supplementary Material**. Further inquiries can be directed to the corresponding author.

## Ethics statement

This study was reviewed and approved by The Ethics Committee in Inner Mongolia People’s Hospital.

## Author contributions

Conceptualization, ZW, TY and LY. Methodology, XH, KL, TW, ZF and XZ. Software, XH, KL and TW. Validation, XH, TW, ZF, MW and XZ. Formal analysis, ZW, XH, ZF, XZ, MW and FL. Investigation, XH, ZF, XZ, MW and FL. Resources, ZW and LY. Data curation, ZW and XH. Writing-original draft preparation, ZW, TY and LY. Writing—review and editing, ZW, XH, TY and LY. Visualization, JJ and WG. Supervision, WG and LY. Project administration, ZW and LY. Funding acquisition, ZW and LY. All authors contributed to the article and approved the submitted version.

## Funding

This work was supported by the Science and Technology Planning Project of Inner Mongolia Science and Technology Department (Grant No. 201802153), Inner Mongolia Natural Science Foundation (Grant No. 2020MS08157), National Natural Science Foundation of China (Grant No. 81960449), and Scientific Research Project Foundation of Inner Mongolia Health Commission (Grant No. 201702003). The work has been supported by the Talent training plan for the Key laboratory of Inner Mongolia Science and Technology Department.

## Conflict of interest

The authors declare that the research was conducted in the absence of any commercial or financial relationships that could be construed as a potential conflict of interest.

## Publisher’s note

All claims expressed in this article are solely those of the authors and do not necessarily represent those of their affiliated organizations, or those of the publisher, the editors and the reviewers. Any product that may be evaluated in this article, or claim that may be made by its manufacturer, is not guaranteed or endorsed by the publisher.

## Supplementary material

SUPPLEMENTARY VIDEO 1 The dynamic procedure of CRISPR/Cas9_-3NLS-AF-488_/sgHMGA2@PDA enteringthe MKN-45 cells.

SUPPLEMENTARY VIDEO 2 The 3D photos of CRISPR/Cas9_-3NLS-AF-488_/sgHMGA2@PDA entering the nuclei.
