# Supplementary material for: CRISPR/Cas9-3NLS/sgHMGA2@PDA nanosystem is the potential efficient gene editing therapy for gastric cancer with HMGA2 high expression
Source: Front Oncol. 2022 Sep 2;12:978533. doi: 10.3389/fonc.2022.978533 (PMC9479195; doi:10.3389/fonc.2022.978533)
Supplement: Supplementary file 3 [file DataSheet_1.pdf]

Supplementary Material

Table S1 Gene editing efficiency of different RNP delivery systems

Delivery system	Cell Type	Efficiency	Refs
**CRISPR/Cas9$_{\text{-3NLS}}$/sgHMGA2@PDA**	**MKN-45 cells**	**82%**	**This work**
Metal organic frameworks	MCF-7 cells	~60%	[1]
Graphene oxide	AGS cells	~39%	[2]
Black phosphorus nanosheets	MCF-7 cells	32.1%	[3]
Calcium phosphate	Protoplast cells	20%	[4]
Gold nanocluster	A375 cells	26.2%	[5]
DNA nanoclews	3T3-L1 cells	75%	[6]
Chitosan nanoparticles	U2OS cells	55.8%	[7]
Poly(β-amino ester) nanoparticles	HEK293T cells	77%	[8]
Lipid nanoparticles	HEK cells	70%	[9]
Lipid nanoparticles	Jurkat T cells	8%	[10]

Amino acid sequences of the CRISPR/Cas9:

10 20 30 40 50

MNFKILPIAI DLGVKNTGVF SAFYQKGTSL ERLDNKNGKV YELSKDSYTL

60 70 80 90 100

LMNNRTARRH QRRGIDRKQL VKRLFKLIWT EQLNLEWDKD TQQAISFLFN

110 120 130 140 150

RRGFSFITDG YSPEYLNIVP EQVKAILMDI FDDYNGEDDL DSYLKLATEQ

160 170 180 190 200

ESKISEIYNK LMQKILEFKL MKLCTDIKDD KVSTKTL**KE**I TSYEFELLAD

210 220 230 240 250

YLANYSESLK TQKFSYTDKQ GNL**KE**LSYYH HDKYNIQEFL KRHATINDRI

260 270 280 290 300

LDTLLTDDLD IWNFNFEKFD FDKNEEKLQN QEDKDHIQAH LHHFVFAVNK

310 320 330 340 350

IKSEMASGGR HRSQYFQEIT NVLDENNHQE GYLKNFCENL HNKKYSNLSV

360 370 380 390 400

KNLVNLIGNL SNLELKPLRK YFNDKIHAKA DHWDEQKFTE TYCHWILGEW

410 420 430 440 450

RVGVKDQDKK DGAKYSYKDL CNELKQKVTK AGLVDFLLEL DPCRTIPPYL

460 470 480 490 500

DNNNRKPPKC QSLILNPKFL DNQYPNWQQY LQELKKLQSI QNYLDSFETD

510 520 530 540 550

LKVLKSSKDQ PYFVEYKSSN QQIASGQRDY KDLDARILQF IFDRVKASDE

560 570 580 590 600

LLLNEIYFQA KKLKQKASSE LEKLESSKKL DEVIANSQLS QILKSQHTNG

610 620 630 640 650

IFEQGTFLHL VCKYYKQRQR ARDSRLYIMP EYRYDKKLHK YNNTGRFDDD

660 670 680 690 700

NQLLTYCNHK PRQKRYQLLN DLAGVLQVSP NFLKDKIGSD DDLFISKWLV

710 720 730 740 750

EHIRGFKKAC EDSLKIQKDN RGLLNHKINI ARNTKGKCE**K E**IFNLICKIE

760 770 780 790 800

GSEDKKGNYK HGLAYELGVL LFGEPNEASK PEFDRKIKKF NSIYSFAQIQ

810 820 830 840 850

QIAFAERKGN ANTCAVCSAD NAHRMQQIKI TEPVEDNKDK IILSAKAQRL

860 870 880 890 900

PAIPTRIVDG AVKKMATILA KNIVDDNWQN IKQVLSAKHQ LHIPIITESN

910 920 930 940 950

AFEFEPALAD VKGKSLKDRR KKALERISPE NIFKDKNNRI **KE**FAKGISAY

960 970 980 990 1000

SGANLTDGDF DGA**KE**ELDHI IPRSHKKYGT LNDEANLICV TRGDNKNKGN
1010 1020 1030 1040 1050
RIFCLRDLAD NYKLKQFETT DDLEIEKKIA DTIWDANKKD FKFGNYRSFI
1060 1070 1080 1090 1100
NLTPQEQKAF RHALFLADEN PIKQAVIRAI NNRNRTFVNG TQRYFAEVLA
1110 1120 1130 1140 1150
NNIYLRAK**KE** NLNTDKISFD YFGIPTIGNG RGIAEIRQLY EKVDSDIQAY
1160 1170 1180 1190 1200
AKGDKPQASY SHLIDAMLAF CIAADEHRND GSIGLEIDKN YSLYPLDKNT
1210 1220 1230 1240 1250
GEVFTKDIFS QIKITDNEFS DKKLVRKKAI EGFNTHRQMT RDGIYAENYL
1260 1270 1280 1290 1300
PILIH**KE**LNE VRKGYTWKNS EEIKIFKGKK YDIQQLNNLV YCLKFVDKPI
1310 1320 1330 1340 1350
SIDIQISTLE ELRNILTTNN IAATAEYYYI NLKTQKLHEY YIENYNTALG
1360 1370 1380 1390 1400
YKKYS**KE**MEF LRSLAYRSER VKIKSIDDVK QVLDKDSNFI IGKITLPFK**K**
1410 1420 1430 1440 1450
EWQRLYREWQ NTTIKDDYEF LKSFFNVKSI TKLHKKVRKD FSLPISTNEG
1460 1470 1480 1490 1500
KFLVKRKTWD NNFIYQILND SDSRADGTKP FIPAFDISKN EIVEAIIDSF
1510 1520 1530 1540 1550
TSKNIFWLPK NIELQKVDNK NIFAIDTSKW FEVETPSDLR DIGIATIQYK
1560 1570 1580 1590 1600
IDNNSRPKVR VKLDYVIDDD SKINYFMNHS LLKSRYPDKV LEILKQSTII
1610 1620 1621
EFESSGFNKT I**KE**MLGMKLA GIYNETSNN

Fig. S1. Amino acid sequences of the CRISPR/Cas9. The KE sequence has been highlighted in yellow.

The sequences are extracted from UniprotKB-A0Q5Y3 (CAS9_FRATN).

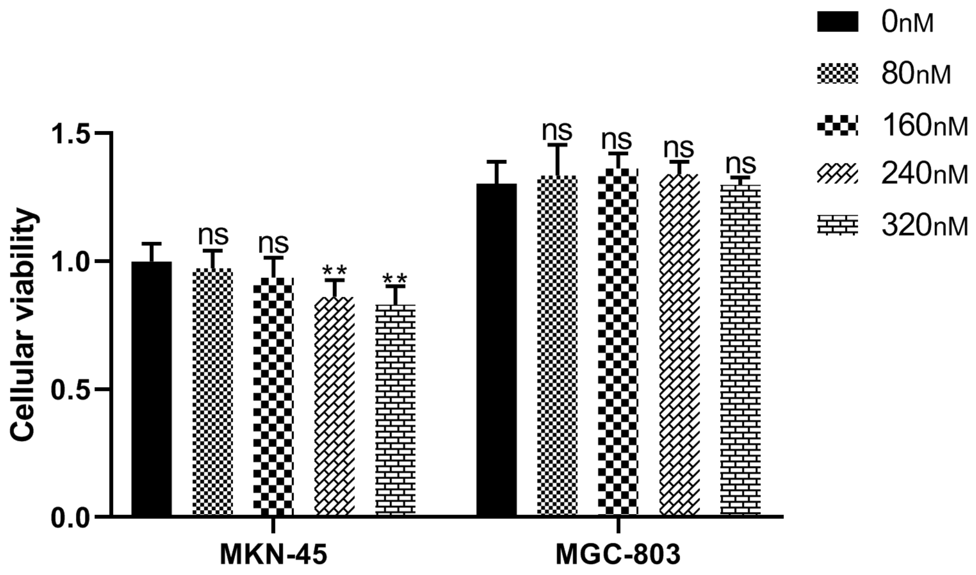

Fig. S2. Cell viability of MKN-45 and MGC-803 cells after incubating with CRISPR/Cas9$_{-3NLS}$@PDA (n=3)，ns: no significant differences; ** *P*< 0.01; **** *P*< 0.001, Student’s t test. Error bars represent SD.

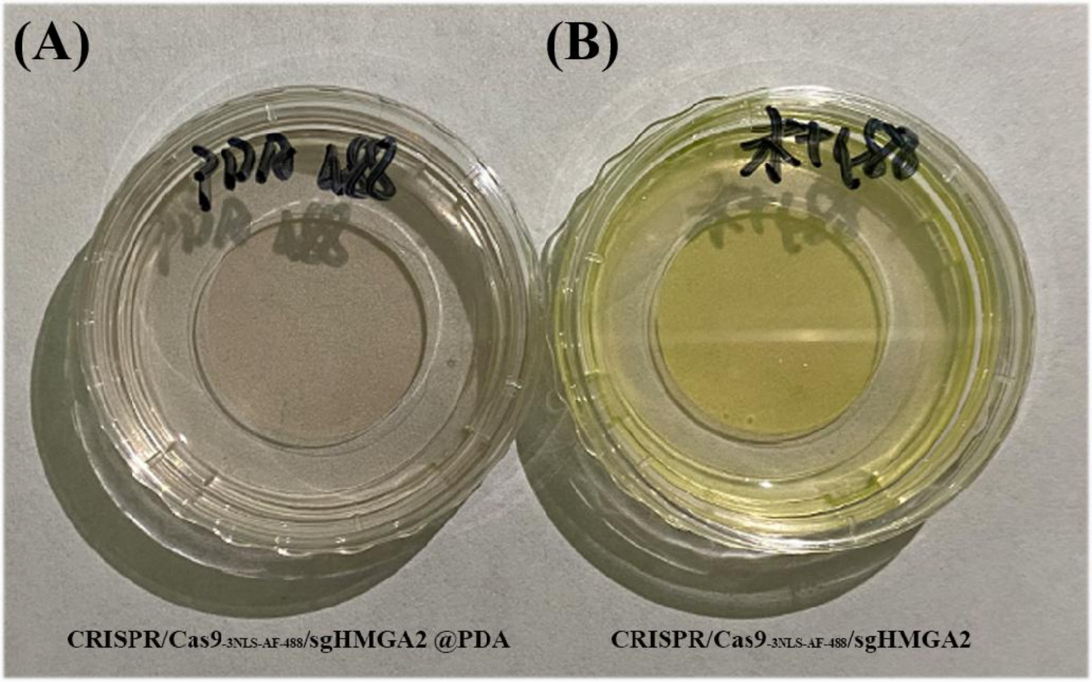

Fig. S3. (A) The color of CRISPR/Cas9-3NLS-AF-488/sgHMGA2@PDA solution. (B) The color of CRISPR/Cas9-3NLS-AF-488/sgHMGA2 solution

References

[1] M.Z. Alyami, S.K. Alsaiari, Y. Li, S.S. Qutub, F.A. Aleisa, R. Sougrat, J.S. Merzaban, N.M. khashab, Cell-Type-Specific CRISPR/Cas9 Delivery by Biomimetic Metal Organic Frameworks, J Am Chem Soc 142 (2020) 1715-1720.

[2] H. Yue, X. Zhou, M. Cheng, D. Xing, Graphene oxide-mediated Cas9/sgRNA delivery for efficient genome editing, Nanoscale 10 (2018) 1063-1071.

[3] W. Zhou, H. Cui, L. Ying, Enhanced Cytosolic Delivery and Release of CRISPR/Cas9 by Black Phosphorus Nanosheets for Genome Editing, Angew Chem Int Edit 57 (2018) 10268-10272.

[4] S. Li, Z. Song, Biomimetic Mineralization-Based CRISPR/Cas9 Ribonucleoprotein Nanoparticles for Gene Editing, ACS Appl Mater Interfaces 11 (2019) 47762-47770.

[5] E. Ju, T. Li, S. Ramos da Silva, S.J. Gao, Gold Nanocluster-Mediated Efficient Delivery of Cas9 Protein through pH-Induced Assembly-Disassembly for Inactivation of Virus Oncogenes, ACS Appl Mater Interfaces 11 (2019) 34717-34724.

[6] W. Sun, J. Wang, CRISPR-Cas12a delivery by DNA-mediated bioresponsive editing for cholesterol regulation, Sci Adv 6 (2020) eaba2983.

[7] J. Qiao, W. Sun, S. Lin, R. Jin, L. Ma, Y. Liu, Cytosolic delivery of CRISPR/Cas9 ribonucleoproteins for genome editing using chitosan-coated red fluorescent protein, Chem Commun 55 (2019) 4707-4710.

[8] Y. Rui, D.R. Wilson, Carboxylated branched poly(β-amino ester) nanoparticles enable robust cytosolic protein delivery and CRISPR-Cas9 gene editing, Sci Adv 5 (2019) eaay3255.

[9] M. Wang, J.A. Zuris, F. Meng, H. Rees, S. Sun, P. Deng, Y. Han, X. Gao, D. pouli, Q. Wu, I. Georgakoudi, D.R. Liu, Q, Xu, Efficient delivery of genome-editing proteins using bioreducible lipid nanoparticles, Proc Natl Acad Sci U S A 113 (2016) 2868-2873.

[10] P. Gee, M.S.Y. Lung, Extracellular nanovesicles for packaging of CRISPR-Cas9 protein and sgRNA to induce therapeutic exon skipping, Nat Commun 11 (2020) 1334.
